# Unilateral Vogt-Koyanagi-Harada Disease: Report of Two Cases

**DOI:** 10.4103/0974-9233.75898

**Published:** 2011

**Authors:** Alok Agrawal, Jyotirmay Biswas

**Affiliations:** Department of Uveitis, Medical Research Foundation, Sankara Nethralaya, Nungambakkam, Chennai, India; 1 Department of Ocular Pathology and Uveitis, Medical Research Foundation and Vision Research Foundation, Sankara Nethralaya, Nungambakkam, Chennai, India

**Keywords:** Choroidal Striae, Disc Edema, Fundus Fluorescein Angiography, Ultrasonography, Vogt-Koyanagi-Harada Disease

## Abstract

In this retrospective report, we present two cases of unilateral Vogt-Koyanagi-Harada (VKH) disease. These patients were evaluated with clinical, ophthalmological and laboratory examinations. Their response following corticosteroid administration was evaluated. Both patients had the characteristic clinical features of VKH involving only one eye, including disc edema, choroidal striae, multiple sub retinal yellow lesions and exudative retinal detachment. These cases indicate that the clinical and angiographic features were typical of VKH disease despite the unilateral involvement.

## INTRODUCTION

Vogt-Koyanagi-Harada (VKH) disease is a multisystem disorder that affects the eye, inner ear, skin and meninges. The characteristic ocular manifestations are severe bilateral panuveitis with iridocyclitis, serous retinal detachment, diffuse choroidal swelling and optic disc hyperemia. These findings are typically bilateral, but the severity may be asymmetric. Reports of unilateral VKH are meagre.^1^–^3^ We report two additional cases of unilateral VKH disease.

## CASE REPORTS

### Case 1

**Figure 1:**
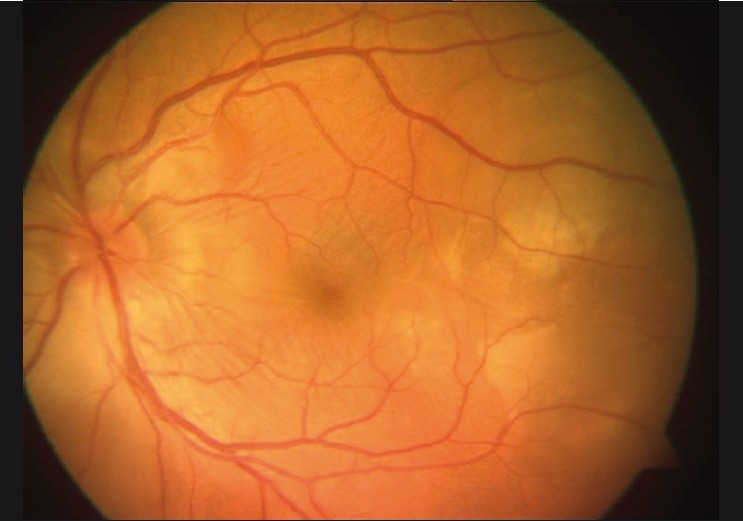
Fundus photo showing disc edema with multifocal serous detachment at the posterior pole in the left eye

**Figure 2:**
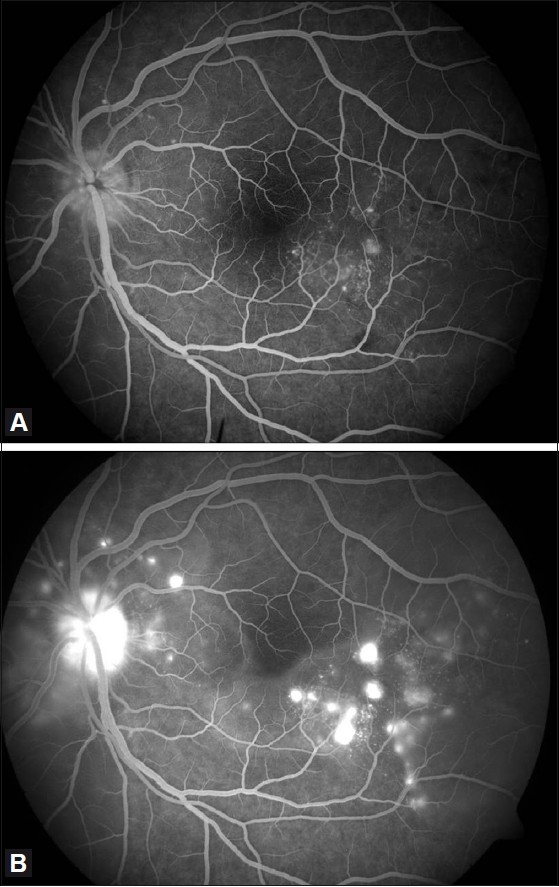
(A) Fundus fluorescein angiography of the left eye showing multiple pinpoint hyperfluorescence in the early arteriovenous phase. (B) Fundus fluorescein angiography of the left eye showing pooling of dye in the subretinal space in the late arteriovenous phase

A 40-year-old male presented with a history of blurring of vision in the left eye for 2 days. He had a similar episode 1 month prior to presentation and consulted a local ophthalmologist who diagnosed subretinal exudates with choroiditis for which he was treated with oral steroids. His vision improved with treatment. The patient had a history of sinusitis 6 months prior to presentation. There was no history of tinnitus, vertigo, headache, neck stiffness, alopecia, trauma or previous ocular surgery. On examination, the best-corrected visual acuity (BCVA) was 20/20, N6 in the right eye and 20/125, N36 in the left eye. The slit-lamp examination (SLE) in the right eye was normal. The left eye had 1+ aqueous flare. There were no vitreous cells and the intraocular pressures (IOP) were 14 mmHg and 32 mmHg in the right and left eyes, respectively. Fundus evaluation of the right eye was normal whereas the left eye had disc edema and choroidal striae with multiple subretinal yellow lesions over the posterior pole Figure 1. A fundus fluorescein angiography (FFA) showed multiple spots of pinpoint hyperfluorescence in the early arteriovenous phase with pooling and leakage of dye in the subretinal space in the late stage of angiogram in the left eye [Figure 2 A and B]. The B-scan ultrasonography measured choroidal thickness of 1.5 mm in the right eye and 1.3 mm in the left eye.

The patient was diagnosed with unilateral VKH disease. The patient was in the glaucoma clinic due to high IOP. Gonioscopy revealed open angles bilaterally. The patient was treated with 0.5% timolol eye drops twice-daily for 1 month in the left eye to lower the IOP. On subsequent visits, his IOP was normal. Laboratory investigations revealed an increased erythrocyte sedimentation rate/rheumatoid arthiritis (ESR/RA) factor, and fluorescent antinuclear antibody was negative. After a physician’s clearance, the patient was administered intravenous methyl prednisolone (IVMP) at 1 g daily for three consecutive days. After 1 week, the vision improved to 20/16, N6 and 20/32, N6 in the right and left eyes, respectively. IOP was normal bilaterally. The fundus in the right eye was normal and the left eye showed regression of the retinal edema, choroidal striae and subretinal lesions. The patient was prescribed oral prednisolone 60 mg once-daily after breakfast everyday on a tapering schedule, with calcium 500 mg and ranitidine 150 mg twice-daily for 6 weeks. After 6 weeks, the BCVA was 20/20, N6, bilaterally. The IOP and the fundii were normal bilaterally. During this visit, the B-scan ultrasonography measured a choroidal thickness of 1.6 mm in the right eye and 1.8 mm in the left eye. These parameters remained stable after 4 months.

## Case 2

**Figure 3:**
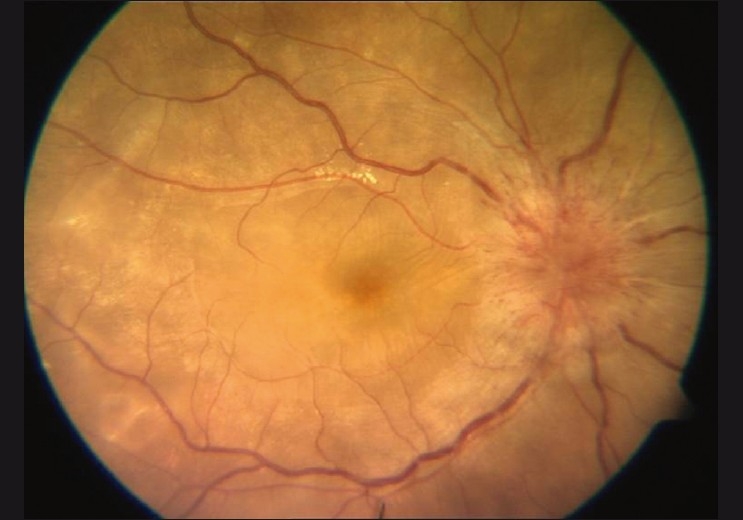
Fundus photo of the right eye showing disc edema with serous retinal detachment at the posterior pole

A 14-year-old male presented with a history of sudden decrease in vision in the right eye for 3 weeks. There was no history of tinnitus, vertigo, headache, neck stiffness, alopecia, trauma or previous ocular surgery. On examination, the BCVA was 20/600, < N36 in the right eye and 20/20, N6 in the left eye. SLE of the right eye revealed a quiet anterior chamber with 1+ vitreous cells, and the left eye was unremarkable. IOP was normal bilaterally. Disc edemas and serous retinal detachment with subretinal precipitates at the posterior pole were present on the fundus examination of the right eye [Figure 3]. The fundus examination of the left eye was normal.

**Figure 4:**
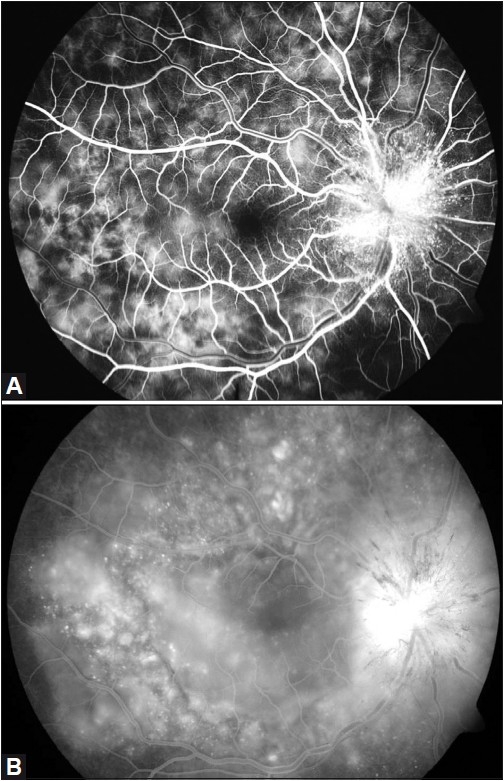
(A) Fundus fluorescein angiography of the right eye showing staining of the optic disc in the early arteriovenous phase with multiple pinpoint hyperfluorescence. (B) Fundus fluorescein angiography of the right eye showing staining of the optic disc with pooling of dye in the subretinal space in the late arteriovenous phase

FFA results included staining of the disc with multiple pinpoint leaks in the early arterio-venous phase [Figure 4A] and pooling of the dye in the subretinal space during the late arterio-venous phase of angiogram in the right eye [Figure 4B]. On B-scan ultrasonography, choroidal thickness was 3.2 mm in the right eye and 1.3 mm in the left eye, respectively. Subretinal fluid with internal limiting membrane striae in the right eye was present on optical coherence tomography and the left eye was unremarkable. Laboratory investigations revealed an increased ESR and RA factor while fluorescent antinuclear antibody was negative. After a physician’s clearance, the patient had IVMP 1 g daily for three consecutive days. The patient was diagnosed with unilateral VKH disease. Magnetic resonance imaging studies of the brain and orbit were normal.

After 3 days, the BCVA was 20/80, N36 and 20/20, N6 in the right and left eyes, respectively. IOP was normal bilaterally. The retina of the right eye was attached. The patient was prescribed immunosuppressive medication tablet Imuran 150 mg thrice-daily for 1 month, twice-daily for 2 months and once-daily for 1 month combined with oral prednisolone 60 mg once everyday after breakfast in tapering doses along with calcium 500 mg and ranitidine 150 mg twice-daily in tapering doses for 6 weeks. After 6 weeks, the BCVA in both eyes was 20/20, N6. IOP was normal bilaterally. The fundus was normal bilaterally.

Three years after treatment, the BCVA in the right eye was 20/20, N6. IOP was normal bilaterally. A macular scar was present in the right eye. The left eye had active multifocal choroiditis. The patient underwent FFA, which showed active lesions in the left eye, and he was subsequently treated with oral predinisolone 60 mg once everyday after breakfast along with calcium 500 mg and ranitidine 150 mg twice-daily in tapering doses for six weeks. On subsequent visits, his eye condition became stable.

## DISCUSSION

The differential diagnosis of VKH includes other causes of posterior uveitis and panuveitis, particularly diseases or conditions such as sympathetic ophthalmia, syphilis intraocular lymphoma, Lyme disease and bilateral diffuse melanocytic hyperplasia that is seen in patients with systemic lupus erythematosus and may present with serous retinal detachment mimicking VKH disease. History of the associated pathology is helpful in ruling out VKH disease. Additionally, in all cases of uveitis, particularly atypical cases, infectious diseases such as syphilis should be ruled out. Testing was not performed in this patient because of financial constraints. The absence of dermatologic manifestations was not surprising as these may not become evident until later in the disease process. Usui *et al*. reported three cases with clinical and laboratory features typical of VKH disease, except for the unilateral involvement.^1^ Forster *et al*. reported one case in a young boy who presented with unilateral disease.^2^ Kouda *et al*. recommended that VKH be considered in the differential diagnosis of cases with posterior scleritis.^3^

Our cases revealed that clinical and angiographic features can be typical of VKH disease, except for the unilateral involvement. It is important for ophthalmologists to recognize unilateral VKH disease even though it is a rare clinical variant of the disease. Our case reports highlight the unusual presentation of two rare cases of unilateral VKH disease.
